# Ranking of Business Process Simulation Software Tools with DEX/QQ Hierarchical Decision Model

**DOI:** 10.1371/journal.pone.0148391

**Published:** 2016-02-12

**Authors:** Nadja Damij, Pavle Boškoski, Marko Bohanec, Biljana Mileva Boshkoska

**Affiliations:** 1 Faculty for Information Studies, Ulica talcev 8, Novo mesto, Slovenia; 2 Department of Systems and Control, Jožef Stefan Institute, Jamova cesta 39, Ljubljana, Slovenia; 3 Department of Knowledge Technologies, Jožef Stefan Institute, Jamova cesta 39, Ljubljana, Slovenia; University of Maribor, SLOVENIA

## Abstract

The omnipresent need for optimisation requires constant improvements of companies’ business processes (BPs). Minimising the risk of inappropriate BP being implemented is usually performed by simulating the newly developed BP under various initial conditions and “what-if” scenarios. An effectual business process simulations software (BPSS) is a prerequisite for accurate analysis of an BP. Characterisation of an BPSS tool is a challenging task due to the complex selection criteria that includes quality of visual aspects, simulation capabilities, statistical facilities, quality reporting etc. Under such circumstances, making an optimal decision is challenging. Therefore, various decision support models are employed aiding the BPSS tool selection. The currently established decision support models are either proprietary or comprise only a limited subset of criteria, which affects their accuracy. Addressing this issue, this paper proposes a new hierarchical decision support model for ranking of BPSS based on their technical characteristics by employing DEX and qualitative to quantitative (QQ) methodology. Consequently, the decision expert feeds the required information in a systematic and user friendly manner. There are three significant contributions of the proposed approach. Firstly, the proposed hierarchical model is easily extendible for adding new criteria in the hierarchical structure. Secondly, a fully operational decision support system (DSS) tool that implements the proposed hierarchical model is presented. Finally, the effectiveness of the proposed hierarchical model is assessed by comparing the resulting rankings of BPSS with respect to currently available results.

## 1 Introduction

A business process (BP) is defined as a collection of related and structured activities with the aim to create outputs that are produced to serve customers’ needs [1]. This is the fundamental differentiator between terms such as BP on one hand and support process, organisational department/unit, etc. on the other. In particular, BPs are considered to be value-added processes that are only executed when added value to the input or the activity from the customer’s perspective can be achieved. BPs represent an essential part of every organisation, regardless of size or industry. The clarity of their definition and regular optimisation is essential for company’s overall success and profitability. The process view looks at the functioning of the company from the customer’s perspective [1, 2]. However, dealing with improper specification of a BP can have significant negative consequences. Introducing new or updating an existing BP without proper analysis may lead to significant deterioration of the company’s performance. Optimisation of BPs requires usage of modelling techniques and simulation tools [3]. The purpose of business process modelling is to develop a model that reflects the organisation and functionality of an existing or new business process and is as such a predecessor to business process simulation that is usually executed by using of business process simulation tools or softwares [1]. BP analysis is usually performed by simulating the BP behaviour under various conditions and potential “what-if” scenarios and/or sensitivity analysis prior to its implementation [4, 5]. Because of the algorithmic structure of a BP, it is generally possible to simulate it with special simulation tools referred to as business process simulations softwares (BPSSs) [6].

BPSS is increasingly being used to address a variety of issues from the strategic management of software development, to supporting process improvements, to software project management training with the scope of analysing narrow focused portions of the life cycle to longer term product evolutionary models with broad organisational impacts [7]. Two main categories of BPSS are the general purpose discrete event simulation tools and business process modelling tools [8]. BPSS therefore can consist of either modelling tools (a graphical modelling environment, built-in simulation objects with defined properties and behaviour, sampling routines, property sheets and visual controls), tools to execute the simulation (a simulation executive to run a model, animated graphic, virtual reality representation and user interaction with the simulation as it runs), tools to support experimentation, optimisation, results interpretation and presentation, or links to other software (links to spreadsheets, databases, ERP systems) [9]. Selecting the most appropriate BPSS is a daunting task due to the vast number of available simulation tools. Since a multitude of, possibly conflicting, criteria influence this decision, the selection problem can be considered as a multi-criteria decision making problem. This paper addresses the problem of BPSS selection by proposing a hierarchical decision model implemented as a decision support system (DSS).

DSS are readily used for the process of software selection, predominantly for ranking purposes [10–17]. The effectiveness of a DSS is determined by the underlying model. In the context of BPSS, there is a set of characteristics that govern the selection of the optimal tool, for instance functionality, reliability, usability, efficiency, maintainability, portability and personalisation [6, 8, 11]. Each of these characteristics comprises several attributes which results into an attribute set with large cardinality. Despite having defined a comprehensive criteria set, the commonly used decision models comprise only a limited set of criteria [14–16, 18]. As a result, the employed DSSs provide rankings which can be considered as incomplete or partial.

Addressing this issue, a new hierarchical multi-criteria decision model is proposed that comprises 17 criteria grouped in logically interconnected segments covering every aspect of a BPSS packages. The newly designed model is employed for ranking of BPSS packages using a combination of DEX and qualitative to quantitative (QQ) methodologies [19].

The contributions of the proposed approach are the following:

- The hierarchical structure divides the large criteria set into smaller subsets thus providing more readable and easier to understand decision problem, allowing humans to achieve consistent decision making.
- The proposed hierarchical model can be easily extended by adding additional criteria groups and placing them accordingly in the model’s structure.
- The hierarchical decision model is implemented in an operational and freely accessible DSS tool.
- In addition to commercially available BPSS tools, this analysis includes open-source BPSS tools that were previously mainly omitted.
- The effectiveness of the proposed model is assessed by comparing the evaluation results with those provided by proprietary tools, such as Gartner’s [20].

Evaluation of software involves attributes that either indicate the presence of features or describe the quality level of their implementation. In both cases, it is easier to evaluate such attributes using a qualitative than quantitative scale. Consequently, the possible DSS solution should be sought in the class of qualitative decision making methods. In qualitative decision making, one may distinguish two groups of methods. The first group includes methods based on interactive questioning procedures for obtaining the decision makers preferences. Typical representatives are MACBETH [21] and ZAPROS [22]. The second group of methods apply the preference disaggregation principle. Typical methods that belong to this group are UTA [23], DRSA [24], Doctus [25] and DEX [19, 26]. From the possible candidates, this analysis is based on DEX methodology extended with the QQ method [27]. It employs the principle of decomposing the problem into smaller and easily understandable decision components. These are used for obtaining simple decisions that are propagated into the DEX tree structure from the bottom to the top of the tree leading to the final decision [28], which is completely in line with the designed hierarchical model structure. DEX has been previously used for building of hundreds of complex models in different areas, including health care, project management, quality and risk assessment, environmental management and many more [29]. In this work, DEX is extended with QQ in order to provide numeric evaluation results, which are used for BPSSs ranking.

The proposed DEX/QQ based DSS model is employed for evaluating 33 currently available BPSS tools. Both open-source and proprietary solutions are considered. The DSS ranking results are compared to currently available software rankings reports [20].

The paper is organised as follows. The description of the attributes characterising a BPSS tool are presented in Section 2. The details about model building using DEX/QQ methodology is presented in Section 3. Finally, Section 4 presents the ranking results of the proposed DSS, by evaluating 33 different BPSS tools.

## 2 Attribute selection

BPSS tools are complex software packages. Therefore, many aspects have to be considered in order to perform a proper evaluation. This analysis focuses on the technical properties of these software tools. Consequently, the evaluation considers several aspects of the tool such as functionality, reliability, usability, efficiency, maintainability, portability and personalisation [6, 8, 11]. The attribute set used in this analysis is a union of the attributes characterising each of these aspects.

From a decision maker’s point of view, the attributes can be divided into two logical groups:

1. technical/functional requirements and
2. usability and personalisation capabilities.

The qualitative evaluation of the attributes from the first group can be obtained from the technical specifications of the corresponding BPSS tool. This evaluation can be performed in a rather impartial manner, since the values of those attributes can be determined empirically. The values of the second group of attributes depend on the user’s preferences.

Due to the large cardinality of the attribute set, it is preferable to group the attributes into smaller categories linked in a hierarchical structure based on their dependencies. Generally, the candidate BPSSs are ranked according to five different categories:

1. statistical facilities
2. experimental capabilities
3. testing capabilities
4. efficiency and
5. visual aspects.

The first four categories belong to the group of technical/functional attributes. Characterising these categories is directly related to the actual capabilities of the simulation tool. On the other hand, the visual aspects represent the personal appeal of the simulation tool and has generally little effect on the actual performance of the tool itself.

### 2.1 Statistical facilities

Statistical facilities of a BPSS tool cover both simulation capabilities as well as the analysis of the simulation results. Each of these two segments have significantly different requirements and therefore they should be analysed separately.

#### Stochastic simulation capabilities

Typical analysis would focus on the most computationally demanding aspects, the most important ones being: random number generation, model parameter estimation and parallel processing capabilities.

In many cases, the execution of a BP is triggered by random events. In order to simulate such events, one has to be able to generate random numbers from a corresponding probability distribution. Notwithstanding the simplicity of the task, in many cases BPSS tools include only a limited set of probability distributions. Therefore it is of utmost practical importance to have rich library of probability distributions or some form of Markov Chain Monte Carlo sampling algorithms.

Similarly like the input, there are cases when the completion of a certain activity of the meta process can have a stochastic nature too. As a result the BP output can be regarded a random variable. For proper analysis of these results a powerful parameter estimation framework is needed.

Finally, an important aspect is the parallel processing capability i.e., executing a parallel discrete-event simulation with restricted common resource. Such environment would allow simulation of concurrent algorithm helping in determining synchronisation issues.

It is hardly expected that all these requirements are met in a single BPSS. In many cases, the simulation tools provide a certain API interfaces that allow the users to introduce their own extensions which can certainly improve the capabilities of the simulation tool. This is particularly true for the open-source solutions.

#### Statistical reporting

The statistical analysis of the results are crucial for proper understanding of the simulated BP. In its simplest form, the results are represented just with mean values, which can be misleading. In order to gain proper insight of the BP outcome, the statistical reporting should also include the calculation of confidence intervals for every simulated output variable, estimation of most likely probability distributions or at least typical moments and/or cumulant values.

### 2.2 Experiment facilities

When simulating a BP, one usually opts for testing various “what-if” scenarios [5]. Therefore it is important to be able to perform various simulations with minimal effort from the user side. Three main attributes characterise the tool’s experiment facilities: the ability to define the “warm-up” period, capability of executing batch runs and automatic determination of the run length.

#### Warm-up period

When performing random number generation, the underlying methods require the so-called “warm-up” time, i.e., the number of samples until the sampling algorithm reaches a steady state. All the samples, prior to that moment, should be discarded since they fail to represent the underlying distribution. The BPSS tool should be capable for incorporating this simulation parameter.

#### Batch runs

Capability of the software tool to run multiple parallel simulations with varying parameters can significantly speed up the simulation process. Such parallel simulations are also known as batch simulation runs [1].

#### Automatic determination of the run length

For terminating simulations the run can be stopped as soon as the terminating event occurs. However, for simulations that enter a steady state, determining the run length is not a straightforward task, since the simulation can run indefinitely. Therefore, the simulation tool should implement some of the many available approaches for detecting a steady state. In such a way, the simulation run length will be kept in a reasonable interval while in the same time providing sufficiently long data sets enabling proper analysis of the BP.

### 2.3 Testing capabilities

Powerful testing environment is of utmost practical importance. Typically, testing and debugging tools include features such as break points, watch points, execution flow control etc. However for the case of BP modelling, this set of features should also include tools for checking the correctness of chain of activity as well as powerful logging and tracing mechanisms.

#### Logic checks

A BP represents a chain of activities which usually includes branching and in some cases loops. Logical checks of such an architecture is helpful for determining dead branches, circularity, consecutiveness etc. These checks belong to the group of graph architecture checks and are useful during the modelling phase. Such architectural errors can be also detected during the simulation phase also, however their early detection can significantly shorten the design time.

#### Logging and debugging capabilities

When performing demanding simulations it is expected to run into difficulties either with the model performance or parameter choice. Resolving these issues can be significantly improved by logging certain simulation values during the simulation itself. This can be achieved by employing well established logging services. In many cases, the logging data proves as a valuable source for bottleneck detection.

### 2.4 Efficiency

The efficiency of the BPSS tool addresses the way the models are build rather than the implementation of the simulation algorithms. In that manner, there are several aspects that are important: the allowed level of details that can be used when building either the process or its activities, the completeness of a library typical activities, capability of simulating various queuing scenarios and robustness of simulation.

#### Level of detail

Many BPs exhibit high level of complexity either in the process architecture itself or in the phenomena described by the encompassed activities. In either case, the BPSS tool should allow sufficiently detailed implementation which will lead to more accurate representation of the real-world BP.

Such a high level of modelling detail is usually achieved by offering high number of prepared activities that can be incorporated into a complex chain. In some other cases this can be achieved by allowing custom scripts to be written describing the underlying activity.

#### Modelling quality

Quality of modelling is directly related to the allowed detail level. It is expected that the BPSS tool includes a certain library of template processes and activities that can be used directly by setting certain set of parameters. When such a library is poorly implemented or non-existing the modelling process will become both time consuming and with degraded quality.

#### Queuing policies

Having predetermined set of queuing policies can significantly improve the quality of the model. In such a case, each activity can be associated with certain strategy of request behaviour that corresponds with the real-world scenarios. In its simplest form, requests will wait for activity execution indefinitely.

#### Robustness

It is expected that the most of the BPs are stateful i.e., there is record of the previous processing that influences any future actions. For such cases it is quite important how the BPSS handles fault recoveries. In the case of complex simulations, the BPSS tool should be able to store the BP state at certain milestones in a non-volatile memory. Afterwards, the fault recovery process would resume from the last checkpoint thus avoiding the need for restarting the simulation from the very beginning.

### 2.5 Visual aspects

The graphical capabilities can help visualising bottlenecks thus providing better understanding of the behaviour of a BP. This category adds to the user-friendly aspect of the BPSS tool.

Apart from the simulation visualisation, animation capabilities can significantly help in the process of debugging. If designed properly, it will allow visualisation of execution paths, current input and output values as well as current property values of the actors in the BP.

## 3 DEX and QQ methodology

The evaluation of BPSS tools is performed by qualitative multi-criteria decision making method called DEX. DEX classifies qualitative multi-attribute alternatives into several qualitative classes [19, 26]. In context of DEX, each BPSS tools represents an alternative that is described by the attributes described in Section 2.

DEX methodology decomposes the complex decision problem at hand into smaller and easily understandable decision components, which are assembled into a hierarchical model. There are two types of attributes in DEX: *basic* and *aggregated* ones. The former are the directly measurable attributes, also called input attributes that are used for describing the alternatives at hand. The latter are obtained by aggregating the basic and/or other aggregated attributes.

**Fig 1:**
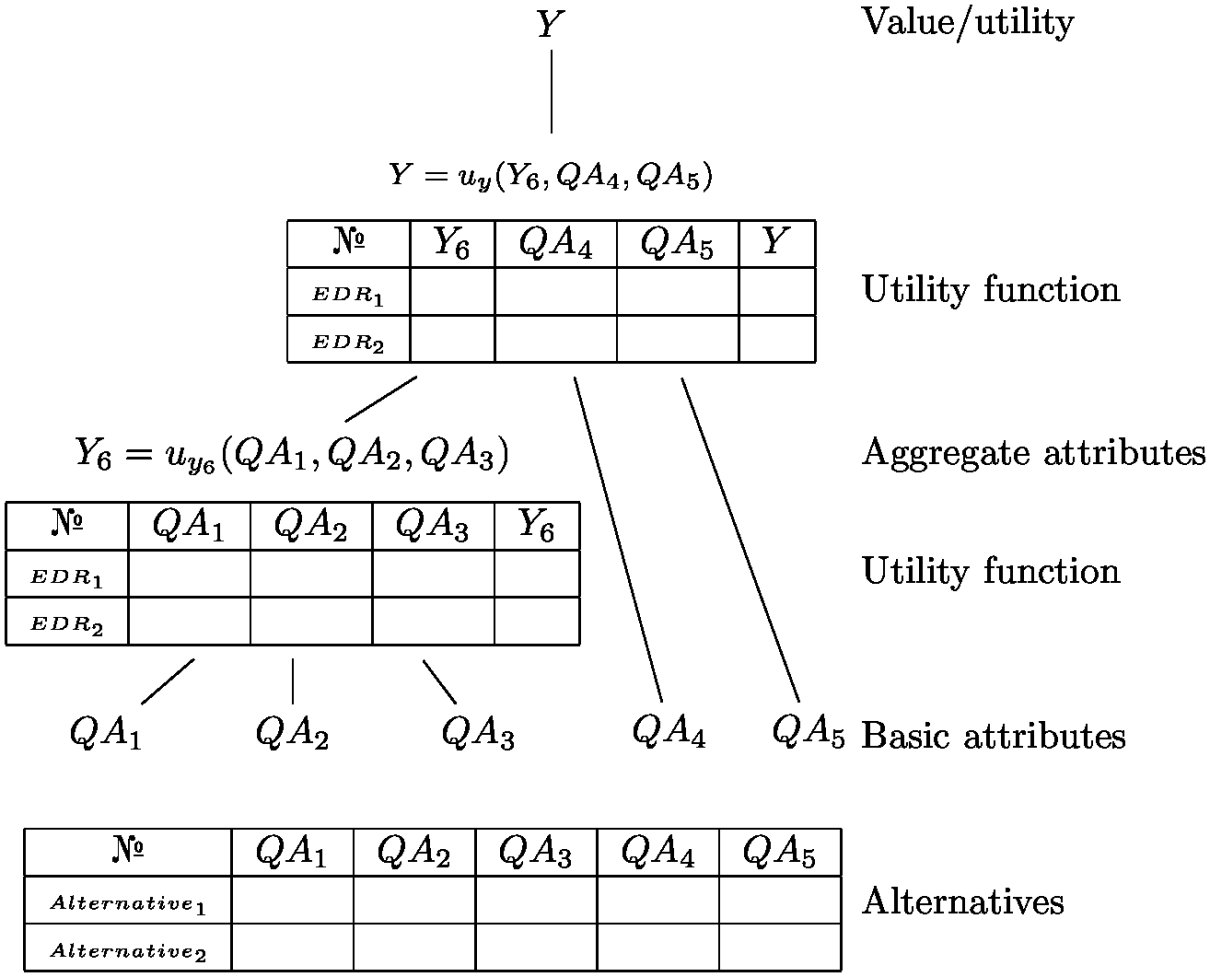
Hierarchical DEX model tree.

The hierarchical attribute structure resembles a tree. The attributes are structured so that there is only one path from each attribute to the root of the tree. The path contains the dependencies among attributes such that the higher-level attributes depend on their immediate descendants in the tree. This structure is shown in Fig 1.

In DEX each of the *n* attributes is a discrete one i.e., the *i*^th^ qualitative attribute *QA*_*i*_, *i* ∈ [1, *n*], can obtain values from a finite value set with *N*_*i*_ elements:

$$\begin{array}{c} QA_{i}\in \left\{e_{1}^{\left(i\right)},e_{2}^{\left(i\right)},\ldots ,e_{N_{i}}^{\left(i\right)}\right\}. \end{array}$$ (1)

The dependency among the attributes is defined by a utility function. For every aggregated attribute *y*, the arguments of the corresponding utility function comprise its immediate descendants from the hierarchical tree. Consequently, the utility function reads as:

$$\begin{array}{c} Y=u_{y}\left(QA_{1},QA_{2},\ldots ,QA_{n}\right). \end{array}$$ (2)

Due to the discrete nature of the attributes, the utility Function (2) is usually represented as a qualitative decision table, as shown in Fig 1 and Table 1. Each row in Table 1 represents an elementary decision rule. Such a rule consists of a subset of attributes and their evaluation into an aggregated attribute. In the decision table, each elementary decision rule can be interpreted as an *if-then* rule.

**Table 1:** Utility Function (2) in a form of a decision table. *L* is the number of elementary decision rules in the table.

№	*QA*_1_	*QA*_2_	⋯	*QA*_*n*_	*Y*
1	*qa*_11_	*qa*_12_	⋯	*qa*_1*n*_	*y*_1_
2	*qa*_21_	*qa*_22_	⋯	*qa*_2*n*_	*y*_2_
⋮	⋮	⋮		⋮	⋮
L	*qa*_*L*1_	*qa*_*L*2_	⋯	*qa*_*Ln*_	*y*_*L*_

**Fig 2:**
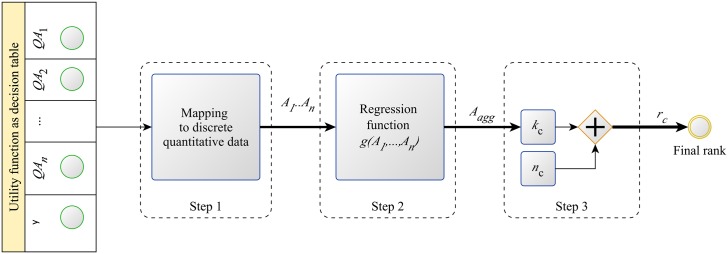
Workflow describing the transformation process from qualitative attributes and quantitative features to final quantitative evaluation.

Elementary decision rules that are almost equally preferred, have the same output *y*_*i*_ and are said to belong to the same qualitative class. Consequently, for DEX these elementary decision rules are indistinguishable. This can be resolved by employing the method QQ method [27]. The three step algorithm describing the DEX/QQ modelling approach is schematically shown in Fig 2.

First, the arguments and the output of the qualitative utility Function (2) are mapped into discrete quantitative ones as:

$$\begin{array}{c} C=U_{c}\left(A_{1},\ldots ,A_{n}\right), \end{array}$$ (3)

where $A_{1},\ldots ,A_{n}\in Z$ (step 1 in Fig 2). The mapping function must preserve the preference order, i.e. the higher the preference of *QA*_*i*_ the greater the value of *A*_*i*_. The goal is to preserve the preference order of attributes, and not the intensity of the preference. Consequently, the ordered qualitative attributes are mapped into ordered discrete values that are equally distanced. The resulting quantitative utility Function (3) is shown in Table 2.

**Table 2:** Utility Function (3) in a form of a decision table. *L* is the number of elementary decision rules and *P* is the number of distinct classes.

№	*A*_1_	*A*_2_	⋯	*A*_*n*_	*C*
1	*a*_11_	*a*_12_	⋯	*a*_1*n*_	*c*_1_
2	*a*_21_	*a*_22_	⋯	*a*_2*n*_	*c*_2_
⋮	⋮	⋮		⋮	⋮
L	*a*_*L*1_	*a*_*L*2_	⋯	*a*_*Ln*_	*c*_*P*_

In the second step, a utility function $g:R^{n}\to R$ is obtained such that

$$\begin{array}{c} A_{agg}=g\left(A_{1},\ldots ,A_{n}\right). \end{array}$$ (4)

The parameter’s values are estimated by employing linear regression. The function defines the relation between the aggregated (dependent) attribute *A*_*agg*_ and input attributes *A*_1_, *A*_2_, …, *A*_*n*_. Despite $A_{i}\in Z$, the function *g*(*A*_1_, …, *A*_*n*_) is defined in $R^{n}$.

The third step ensures consistency between the qualitative and quantitative models. It means that if an elementary decision rule belongs to a quantitative class *c*_*i*_, *i* ∈ {1, …, *P*}, then the output utility value must be in the interval *c*_*i*_ ± 0.5. Additionally it improves the understandability of the quantitative model as it directly shows to which class a certain elementary decision rule belongs to. Therefore, for the utility Function (4), a set of functions *f*_*c*_*i*__ is defined that ensures compliance with the original class *c*_*i*_ as:

$$\begin{array}{c} f_{c_{i}}\left(A_{1},\ldots ,A_{n}\right)=k_{c_{i}}g\left(A_{1},\ldots ,A_{n}\right)+n_{c_{i}}, \end{array}$$ (5)

where *k*_*c*_*i*__ and *n*_*c*_*i*__ are calculated as:

$$\begin{array}{c} k_{c_{i}}=\frac{1}{max_{c_{i}}-min_{c_{i}}} \end{array}$$ (6)

$$\begin{array}{c} n_{c_{i}}=c_{i}+0.5-k_{c_{i}}min_{c_{i}}, \end{array}$$ (7)

and *max*_*c*_*i*__ and *min*_*c*_*i*__ are the maximum and minimum value of the function *g*(*A*_1_, …, *A*_*n*_) for a class *c*_*i*_.

The last two steps are presented with Algorithm 1 [28].

**Algorithm 1** Algorithm steps 2 and 3

1: *U*_*C*_ ← {*sort rows*(*U*_*c*_, ∀*c*)}   ▷ sort rows in utility table *U*_*C*_ Eq (3) according to class (output) *C* in ascending order to prepare for step 3 in QQ

2: *Agg* = *g*(*A*_1_, …, *A*_*n*_)   ▷ find the utility function *g* that best describes the set of alternatives *A*_*i*_

3: **for** ∀*el*_*i*_ ∈ *c*_*i*_ where *el*_*i*_ = (*A*_1*i*_, *A*_2*i*_, ⋯ *A*_*ni*_) **do**  ▷ for each elementary decision rule *el*_*i*_ that belongs to class *c*_*i*_

4:   *G*_*c*_*i*__ ← {*g*(∀*A*_*ji*_ ∈ *el*_*i*_ ± 0.5)}  ▷ find all values of *g* for all combinations of *A*_*ji*_ ± 0.5

5:   *min*_*c*_*i*__ = min(*G*_*c*_*i*__)     ▷ find the minimum of the function *g* in class *c*_*i*_

6:   *max*_*c*_*i*__ = max(*G*_*c*_*i*__)     ▷ find the maximum of the function *g* in class *c*_*i*_

7:   $k_{c_{i}}=\frac{1}{max_{c_{i}}-min_{c_{i}}}$        ▷ calculate the coefficient *k*_*c*_*i*__

8:   *n*_*c*_*i*__ = *c*_*i*_ + 0.5 − *k*_*c*_*i*__ *max*_*c*_*i*__        ▷ calculate the coefficient *n*_*c*_*i*__

9: **end for**

## 4 Results and Discussion

### 4.1 DEX hierarchical model for selection of BPSS tool

**Fig 3:**
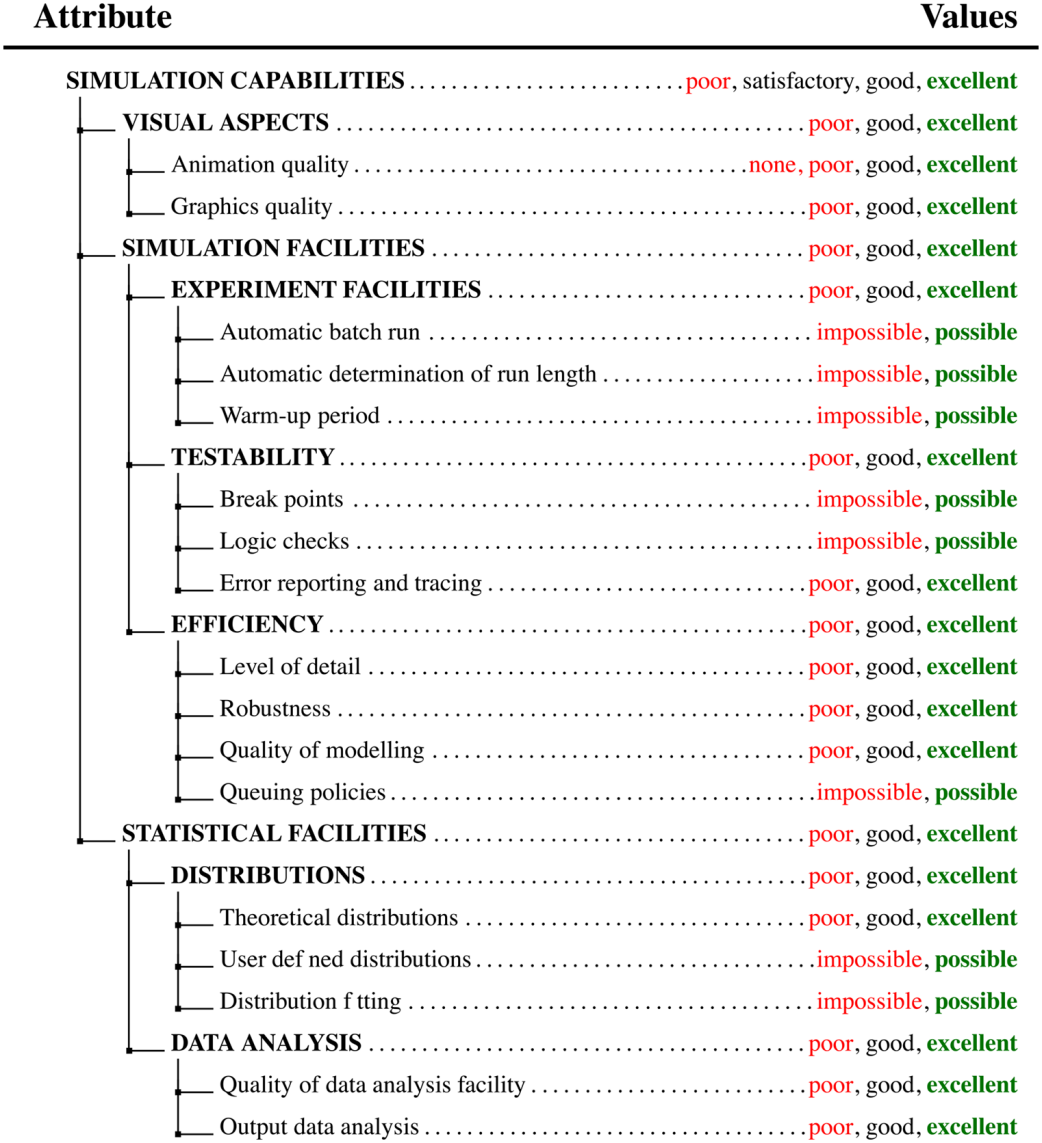
DEX model attributes.

For the problem of choosing the optimal BPSS tool, the hierarchical tree of the proposed DEX model is shown in Fig 3. The structure of the DEX model has been obtained by formulating the characteristics from section 2 in the form of hierarchically structured qualitative attributes. In the presented DEX model, the basic attributes are given with plain letters, while the aggregated attributes are shown with capital letters. For example, in the proposed DEX model, the **SIMULATION CAPABILITIES** of the evaluated BPSS tool are obtained by aggregating the values of the attributes **VISUAL ASPECTS**, **SIMULATION FACILITIES** and **STATISTICAL FACILITIES**. Similarly, the values of the aggregated attribute **SIMULATION FACILITIES** are obtained with aggregation of the values of the attributes **EXPERIMENT FACILITIES**, **TESTABILITY** and **EFFICIENCY**, and so on. The discrete value set Eq (1) is given alongside the corresponding attribute.

As the tree contains nine aggregated attributes, there are nine corresponding utility functions of the form Eq (2). One such utility function, for the attribute **VISUAL ASPECTS (VA)**, is presented in Table 3. From the hierarchical tree (Fig 3), it is clearly visible that the values of the the attribute **VISUAL ASPECTS** are obtained with aggregation of the values of the basic attributes *Animation quality (AQ)* and *Graphics quality (GQ)*. Therefore, the corresponding utility function has the following form:

$$\begin{array}{c} y_{\text{VA}}=u_{y}\left(QA_{AQ},QA_{GQ}\right). \end{array}$$ (8)

The decision table for the utility Function (8) is given with Table 3. In the context of *if-then* rules, the 8^th^ row of the Table 3 can be read as:

$$\begin{array}{c} IFanimation\;quality\;\text{is}\;\text{excellent}\;AND\;Graphics\;quality\;\text{is}\;\text{good}\\ \;THEN\;\text{VISUAL}\;\text{ASPECTS}\;\text{are}\;\text{good}. \end{array}$$

**Table 3:** Utility function for the attribute **VISUAL ASPECTS**.

№	Animation quality	Graphics quality	VISUAL ASPECTS
1	none	poor	poor
2	poor	poor	poor
3	good	poor	poor
4	excellent	poor	good
5	none	good	poor
6	poor	good	good
7	good	good	excellent
8	excellent	good	excellent
9	none	excellent	poor
10	poor	excellent	good
11	good	excellent	excellent
12	excellent	excellent	excellent

The utility function presented in Table 3 defines a partial ranking. For example, for the 11^th^ and the 12^th^ elementary decision rule, the utility function obtains value **excellent**. However it is clear that the 12^th^ elementary decision rule (EDR) is better than the 11^th^ elementary decision rule since the attribute *Animation quality* acquires higher value for the 12^th^ elementary decision rule.

This issue can be resolved by applying the QQ method. As a result, the original utility function presented in Table 3 is transformed into the one shown in Table 4. The mapped discrete quantitative values in Table 4 are employed for fitting the aggregating Function (4). The final rank (the last column in Table 4) is calculated by employing Eq (5). Each linear function in Eq (5) represents a model for the corresponding class in the originally defined qualitative decision table. These functions are used to rank the options in the classes.

**Table 4:** From qualitative to quantitative evaluations using QQ method. The complete data-set is given in S1 DSS Implementation.

№	Animation quality	Graphics quality	VISUAL ASPECTS	Evaluation
1	1	1	1	0.72
2	2	1	1	0.91
3	3	1	1	1.09
4	4	1	2	1.28
5	1	2	1	0.97
6	2	2	2	1.82
7	3	2	3	2.75
8	4	2	3	2.96
9	1	3	1	1.22
10	2	3	2	2.18
11	3	3	3	3.04
12	4	3	3	3.25

For the elementary decision rules given in Table 4, the Eq (4) has the form:

$$\begin{array}{c} g=0.833A_{1}+0.500A_{2}-0.778. \end{array}$$ (9)

For the same table the functions *f*_*c*_*i*__ Eq (5) reads as follows:

$$\begin{array}{c} f_{c_{i}}=\{\begin{array}{ccc} & 0.4013g+0.5151, & \text{if}\;i=1;\\ & 0.5825g+0.8859, & \text{if}\;i=2;\\ & 0.4580g+1.8034, & \text{if}\;i=3. \end{array} \end{array}$$ (10)

### 4.2 Ranking results

For validation purposes, a set of 120 BPSS tools was identified. The list contained both proprietary as well as open-source tools. From the initial 120 tools, 33 were available either as a demo installation or as a completely operating tool. The latter group predominantly consists of open-source tools. Each of these tools was installed and evaluated. In cases for which the demo installations offered only limited capabilities, the technical data sheets were used in order to determine the appropriate evaluation grade for a particular attribute. The validation set was limited to 33 alternatives, since for the remaining 87 tools, it was impossible to determine the appropriate values for a substantial number of attributes.

It has to be noted that the selected set of 33 alternatives was chosen solely for the purpose of evaluating the proposed hierarchical decision model. By selecting an already analysed subset of BPSS tools, the effectiveness of the developed decision model can be directly compared to the results from previously performed analysis.

The complete data-set and the accompanying implementation of the hierarchical model in a working DSS are given in S1 DSS Implementation.

#### Validation

**Fig 4:**
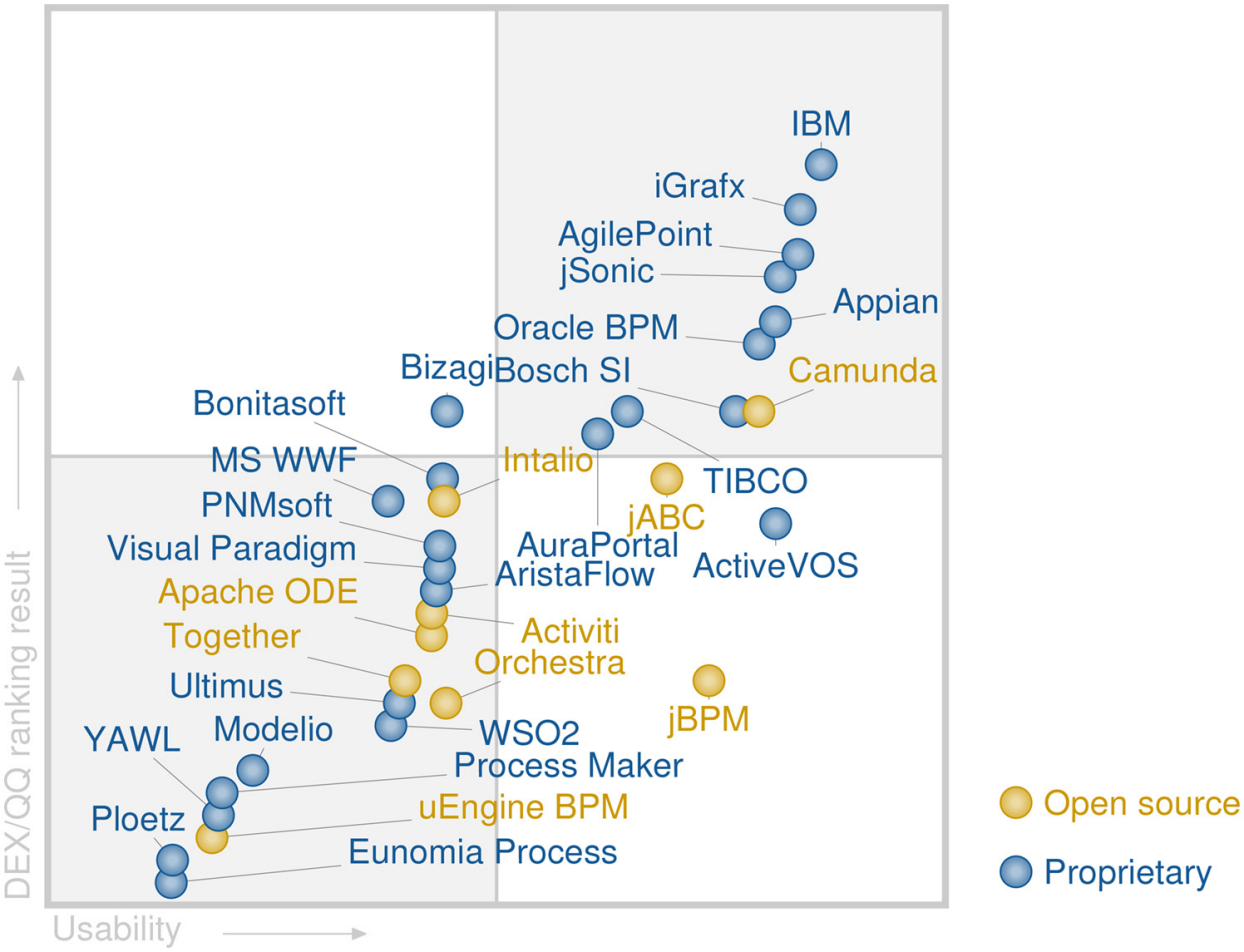
Magic quadrant for BPSS using the proposed hierarchical decision model.

The results from the Gartner [20, 30] analysis are used as a reference. Since Gartner’s results are presented in a form of so-called “magic quadrant”, for comparison purposes, the obtained ranking results are presented in the same form as shown in Fig 4. The term magic quadrant comes from the Gartner’s magic quadrants which use two-dimensional matrix that evaluates vendors based on their completeness of vision and ability to execute. In Fig 4, the values on the ordinate represents the ranking output of the DEX/QQ model. The abscissa is inversely proportional to the effort required for setting up the BPSS tool and performing simple simulation. This is a somewhat personal grade of the required computer skills in order to set up and use a particular BPSS tool, assessed by one of the authors while setting up the software tools.

It should be noted that the proposed DEX/QQ model uses different attributes than Gartner’s. Consequently, the implemented decision rules differ. Nevertheless, the comparison of the locations of the proprietary business process simulations softwares (BPSSs) tools, presented in this as well as in Gartner’s analysis [20, 30], shows that the proposed hierarchical decision model provides similar results. Minor discrepancies are present only in those BPSS tools that are near the borders between two quadrants. Therefore, despite the differences in the decision rules, the results show that the proposed hierarchical decision model provides viable results, compatible to the results from Gartner’s analysis.

It can be noticed that the open source solutions are ranked quite high (values on the abscissa) predominantly due to the novelty and flexibility of the implemented features. However, in many cases, exploitation of the open-source solutions requires significant software skills making them unpopular for broad applicability, hence low values on the ordinate. However, there are cases, like the Camunda package, that despite being open-source, have high “Usability” value. This is mainly due to the support that is offered by the governing company.

#### Discussion on the ranking results

**Fig 5:**
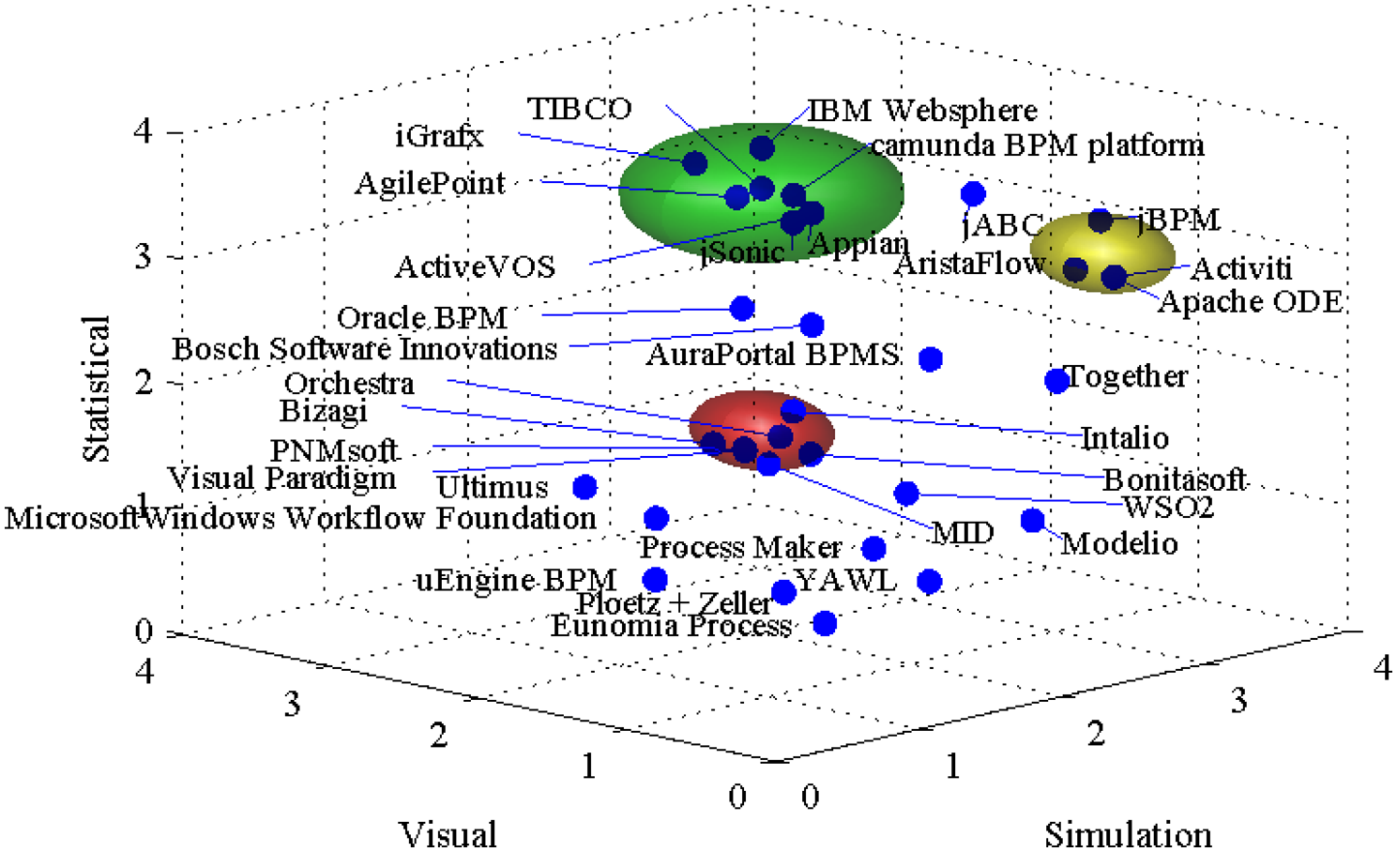
Multi-dimensional representation of the ranking results.

The hierarchical decision model allows an in-depth analysis of the reasons for particular ranking values of a BPSS tool. As shown in Fig 5, there are three typical clusters of BPSS tools. The top ones, marked with green, have the highest marks for all segments. The middle ones, marked with yellow, have poor or none visual functionalities. The last ones, marked with red, lack advanced statistical tools. This clustering reflects the state on the BPSS tools market.

The tools in the yellow cluster are open-source BPSS tools. These business process simulations softwares (BPSSs), in many cases, have the most advanced capabilities for complex simulation and advanced statistical analysis that are even further enhanced with the possibility of adding custom extensions. On the other hand, open-source tools have poor or none visual (or user friendly) properties. In many cases substantial computer skills are required in order to utilise the offered capabilities.

The red cluster includes predominantly proprietary BPSS tools. These tools focus on the user friendly aspects. However, they typically fail to provide sufficiently powerful statistical analysis platforms. This is even further limited by the inability for adding 3^rd^ party extensions.

Therefore, open-source tools tend to have the newest implemented algorithms while in the same time lacking appealing user interfaces. On the other hand, there are many proprietary tools that are user-friendly but offer suboptimal implementations of the crucial simulation capabilities.

### Possible extensions of the model

The hierarchical structure, as shown in Fig 3, can be easily extended by either adding new attributes as leaves in the tree structure or by adding a complete new subtree. The addition of a new attribute would require an extension of the corresponding utility function, i.e., adding an additional attribute *A*_*n*+1_ in the Function (4).

The procedure for adding an additional subtree structure is somewhat more complicated and requires three steps:

**Step 1**: Specify the new utility function in a form of Table 2.
**Step 2**: Convert the qualitative table into a quantitative one using Algorithm 1.
**Step 3**: Extend the utility functions that aggregate the new attributes by adding an additional argument *A*_*n*+1_ to the appropriate functions defined by Eq (4).

A typical extension would be adding a new subtree describing the economical impact of purchasing and running the BPSS tool.

## 5 Conclusion

This study provides a hierarchical decision support model for ranking and choosing of BPSS tools based on DEX/QQ methodology. Such a model enables systematic criteria management due to its capability for incorporating a large number of possibly conflicting criteria as well as fairly simple integration of new ones. Furthermore, the usage of hierarchical decision models leads to simple and focused utility functions thus allowing humans to achieve consistent decision making.

The proposed hierarchical model includes a set of criteria covering all aspects of a BPSS tools performance. The ranking results are inline with the publicly available results for BPSS tools ranking, thus justifying the applicability of the hierarchical decision model in combination with DEX/QQ methodology. Finally, the model is implemented as a freely accessible DSS tool.

## Supporting Information

S1 DSS Implementation MATLAB implementation of the hierarchical decision model with DEX/QQ. The archive contains all the necessary supporting files for DEX/QQ evaluation. The main executable file is DEX_BPSS.m. The experts preferences for each of the categories, shown in Fig 3, are given in DEX_BPSS.mat or in text format dexi.csv. After executing the DEX_BPSS.m script, the final ranking will be stored in the variable final_rank. The data in final_rank can be employed for drawing Figs 4 and 5.
(ZIP)

## References

[1] Damij N, Damij T. A Multi-disciplinary Guide to Theory, Modeling, and Methodology. Progress in IS. Berlin Heidelberg: Springer; 2014.

[2] Damij N, Damij T, Grad J, Jelenc F. A methodology for business process improvement and IS development. Information and software technology. 2008;50:1127–1141. 10.1016/j.infsof.2007.11.004

[3] Laguna M, Marklund J. Business Process Modeling, Simulation, and Design. New Jersey: Pearson Education, Inc; 2005.

[4] Patig S, Stolz M. A pattern-based approach for the verification of business process descriptions. Information and Software Technology. 2013;55(1):58–87. Special section: Best papers from the 2nd International Symposium on Search Based Software Engineering 2010.

[5] Savén R, Olhager J. Integration of product, process and functional orientations: principles and a case study In: Jagdev H, Wortmann J, Pels H, editors. Collaborative Systems for Production Management. vol. 129 of IFIP—The International Federation for Information Processing. Springer US; 2003 p. 375–389.

[6] Rücker B. Building an open source Business Process Simulation tool with JBoss jBPM. Stuttgart University of applied science; 2008.

[7] Kellnera M, Madachyb RJ, Raffoc DM. Software process simulation modeling: Why? What? How? Journal of Systems and Software. 1999;46(2–3):91–105. 10.1016/S0164-1212(99)00003-5

[8] Bosilj-Vuksic V, Ceric V, Hlupic V. Criteria for the Evaluation of Business Process. Interdisciplinary Journal of Information, Knowledge, and Management. 2007;2:73–88.

[9] Pidd M, Carvalho A. Simulation software: Not the same yesterday, today or forever. Journal of Simulation. 2007;1:7–20. 10.1057/palgrave.jos.4250004

[10] Tüzün E, Tekinerdogan B, Kalender ME, Bilgen S. Empirical evaluation of a decision support model for adopting software product line engineering. Information and Software Technology. 2015;60(0):77–101.

[11] Jadhav AS, Sonar RM. Evaluating and selecting software packages: A review. Information and Software Technology. 2009;51(3):555–563. Available from: http://www.sciencedirect.com/science/article/pii/S0950584908001262 10.1016/j.infsof.2008.09.003

[12] Lin HY, Hsu PY, Sheen GJ. A fuzzy-based decision-making procedure for data warehouse system selection. Expert Systems with Applications. 2007;32(3):939–953. Available from: http://www.sciencedirect.com/science/article/pii/S0957417406000509 10.1016/j.eswa.2006.01.031

[13] Azadeh A, Shirkouhi SN, Rezaie K. A robust decision-making methodology for evaluation and selection of simulation software package. The International Journal of Advanced Manufacturing Technology. 2010;47(1–4):381–393. Available from: 10.1007/s00170-009-2205-6 10.1007/s00170-009-2205-6

[14] Jansen-Vullers M, Netjes M. Business process simulation–a tool survey. In: Workshop and Tutorial on Practical Use of Coloured Petri Nets and the CPN Tools; 2006.

[15] Jahangirian M, Eldabi T, Naseer A, Stergioulas LK, Young T. Simulation in manufacturing and business: A review. European Journal of Operational Research. 2010;203(1):1–13. Available from: http://www.sciencedirect.com/science/article/pii/S0377221709004263 10.1016/j.ejor.2009.06.004

[16] Kaschek R, Pavlov R, Shekhovtsov VA, Zlatkin S. Characterization and Tool Supported Selection of Business Process Modeling Methodologies In: Abramowicz W, Mayr H, editors. Technologies for Business Information Systems. Springer Netherlands; 2007 p. 25–37.

[17] Ding S, Xia CY, Zhou KL, Yang SL, Shang JS. Decision Support for Personalized Cloud Service Selection through Multi-Attribute Trustworthiness Evaluation. PLoS ONE. 2014 6;9(6):e97762 Available from: http://dx.doi.org/10.1371%2Fjournal.pone.0097762 10.1371/journal.pone.0097762 24972237 PMC4074036

[18] Changyun L, Haiyan C, Gexin M, Zhibing W. A BPM Software Evaluation Method. In: Intelligent System Design and Engineering Application (ISDEA), 2012 Second International Conference on; 2012. p. 1–4.

[19] Bohanec M, Rajkovic V. DEX: An expert system shell for decision support. Sistemica. 1990;1:145–157.

[20] Norton D, Blechar M, Jones T. Magic Quadrant for Business Process Analysis Tools. Gartner RAS Core; 2010.

[21] Bana e Costa CA, De Corte J, Vansnick J. MACBETH In: Meskens N, Roubens M, editors. Advances in Decision Analysis, Book Series: Mathematical Modelling: Theory and Applications. Kluwer Academic Publishers; 1999 p. 131–157.

[22] Larichev OI. Ranking multicriteria alternatives: The method ZAPROS III. European Journal of Operational Research 131. 2001;131:550–558. 10.1016/S0377-2217(00)00096-5

[23] Jacquet-Lagreze E, Siskos Y. Preference disaggregation: 20 years of MCDA experience. European Journal of Operational Research. 2001;130:233–245. 10.1016/S0377-2217(00)00035-7

[24] Greco S, Matarazzo B, Slowinski R. Rough sets theory for multicriteria decision analysis. European Journal of Operational Research. 2001;129:1–47. 10.1016/S0377-2217(00)00167-3

[25] Baracskai Z, Dörfler V. Automated Fuzzy-Clustering for Doctus Expert System. In: Paper presented at International Conference on Computational Cybernetics, Siófok, Hungary; 2003.

[26] Bohanec M. DEXi: Program for Multi-Attribute Decision Making: User’s manual: version 5.00. IJS Report DP-11897, Jožef Stefan Institute, Ljubljana; 2015.

[27] Bohanec M, Urh B, Rajkovič V. Evaluation of options by combined qualitative and quantitative methods. Acta Psychologica. 1992;80:67–89. 10.1016/0001-6918(92)90041-B

[28] Boshkoska BM. From Qualitative to Quantitative Evaluation Methods in Multi-Criteria Decission Models. Jožef Stefan International Postgraduate School; 2013.

[29] Bohanec M. Statistical Analysis of Qualitative Multi Criteria Decision Models, Developed with Qualitative Hierarchical Method DEX. In: 23rd International Conference on Multiple Criteria Decision Making; 215.

[30] Sinur J, Hill JB. Magic Quadrant for Business Process Management Suites. Gartner RAS Core; 2010.

